# The importance of international collaboration for rare diseases research: a European perspective

**DOI:** 10.1038/gt.2017.29

**Published:** 2017-07-27

**Authors:** D Julkowska, C P Austin, C M Cutillo, D Gancberg, C Hager, J Halftermeyer, A H Jonker, L P L Lau, I Norstedt, A Rath, R Schuster, E Simelyte, S van Weely

**Affiliations:** 1 Department of Health & Biology, Agence Nationale de la Recherche, Paris, France; 2 National Center for Advancing Translational Sciences (NCATS), US National Institutes of Health (NIH), Bethesda, MD, USA; 3 Directorate Health, Directorate-General for Research and Innovation, European Commission, Brussels, Belgium; 4 Directorate Health Systems, Medical products and innovation, Directorate-General for Health and Food Safety, European Commission, Brussels, Belgium; 5 IRDiRC Scientific Secretariat, Inserm US14, Paris, France; 6 Inserm US14-Orphanet, Paris, France; 7 DLR Project Management Agency, Health, Bonn, Germany; 8 The Netherlands Organisation for Health Research and Development (ZonMw), The Hague, The Netherlands

## Abstract

Over the last two decades, important contributions were made at national, European and international levels to foster collaboration into rare diseases research. The European Union (EU) has put much effort into funding rare diseases research, encouraging national funding organizations to collaborate together in the E-Rare program, setting up European Reference Networks for rare diseases and complex conditions, and initiating the International Rare Diseases Research Consortium (IRDiRC) together with the National Institutes of Health in the USA. Co-ordination of the activities of funding agencies, academic researchers, companies, regulatory bodies, and patient advocacy organizations and partnerships with, for example, the European Research Infrastructures maximizes the collective impact of global investments in rare diseases research. This contributes to accelerating progress, for example, in faster diagnosis through enhanced discovery of causative genes, better understanding of natural history of rare diseases through creation of common registries and databases and boosting of innovative therapeutic approaches. Several examples of funded pre-clinical and clinical gene therapy projects show that integration of multinational and multidisciplinary expertize generates new knowledge and can result in multicentre gene therapy trials. International collaboration in rare diseases research is key to improve the life of people living with a rare disease.

## Introduction

A rare disease (RD) is defined as such by the fact that it affects a small percentage of the population. However, there is no internationally recognized definition of a RD. In the European Union (EU), a disease is defined as rare when it is life-threatening or chronically debilitating and when no more than 1 in 2000 people has it.^1^ In the United States, the Rare Diseases Act of 2002^2^ defines a RD strictly according to prevalence, specifically ‘any disease or condition that affects fewer than 200 000 people in the United States’, or ~1 in 1500 people. In Japan, the legal definition of a RD is one that affects fewer than 50 000 patients in Japan, or ~1 in 2500 people.^3^ However, while individually these diseases may be considered rare, collectively they are not. It is estimated that 8% of people have one of the 6000–8000 RDs, and ~36 million Europeans are affected by one or will be. Eighty per cent of RDs are genetic in origin and often result from a dysfunction of a particular pathway (such as a defective gene or protein). Thus, understanding the impact of a single defect can yield insights into more complex pathways and, as result, RDs can serve as models for more common conditions, in particular in the context of precision medicine.

In 2008, the European Commission (EC) adopted a communication^4^ setting out an overall Community strategy to support Member States in diagnosing, treating and caring for EU citizens with RDs. Three main areas were identified: improving recognition and visibility of RDs, supporting national plans for RDs in the EU Member States, and strengthening co-operation and co-ordination for RDs research at the European level. Indeed, RDs are a challenge too big for any one country or world region to tackle alone due to their low prevalence. Research on RDs is not only limited, but also scattered across laboratories and clinics throughout the world. This scarcity of expertize and practice translates into delayed diagnosis, few medicinal products and difficult access to care. That is why the field of RD research is a prime example of a research area that substantially benefits from co-ordination on a European and international scale, as co-operation can bring forth the development of new diagnostics and treatments. By increasing the number of patients available for each study and bringing together the scattered specialists with complementary expertize, the development of new diagnostics and treatments can be notably accelerated. This paper focuses on the efforts in the RDs field in Europe with some additional insights into international activities through the perspective of the International Rare Diseases Research Consortium (IRDiRC).

## Promoting international collaboration on rare diseases in Europe

### EU support schemes

The EU has been funding cross-border research on RD for more than two decades through its Framework Programmes for Research and Technological Development (FP) with complementary projects and actions. Since 1998, more than €1 billion has been invested in over 270 projects on RD (Supplementary Table 1), bringing together a vast array of know-how, experts and resources to improve our understanding of RD in order to develop new diagnostics and therapies for patients, but also to promote best practices used in hospitals and healthcare systems. The participation of small and medium-sized enterprises (SMEs) was encouraged as a way of stimulating European innovation, as RD offer industry the opportunity of developing ‘niche’ markets. Today, supporting international collaboration in RD research remains among the strategic priorities of the EU research funding program Horizon 2020.^5^ The EU continues to offer funding opportunities for RD pre-clinical and clinical research and to strengthen international collaboration, in particular, in view of supporting the development of new therapies for RD and the diagnostic characterization of RD thus to develop better and faster means of high quality and clinical utility for the correct diagnosis of undiagnosed RD.

### European Reference Networks for rare diseases and complex conditions

Set-up under the 2011 Directive on Patient Rights in Cross-Border Healthcare,^6^ European Reference Networks (ERNs) are virtual networks bringing together medical specialists across Europe to tackle rare or complex diseases and conditions that require highly specialized healthcare and a concentration of knowledge and resources. For the first time, a formal structure of voluntary collaboration between healthcare providers across the EU has been created for the direct benefit of the patient. The first 24 thematic networks include over 900 highly specialized healthcare units located in more than 300 hospitals of 25 EU countries plus Norway, and cover a wide range of disease groups including bone disorders, hematological diseases, pediatric cancers and immunodeficiencies; these ERNs will become operational over the course of 2017.^7^

Healthcare providers who are members of ERNs will be connected through a dedicated IT platform and, using a variety of telemedicine tools, offer access to expertize and knowledge of multidisciplinary teams, enabling patients suffering from such conditions to receive the best advice for treatment and diagnosis. A fundamental principle of the ERNs is the stipulation that knowledge should travel rather than patients (with the exception of few cases where patients may be referred for treatment in another country).

Research will be a key element of the ERNs providing a structured framework for joining research efforts across countries, thereby creating a knowledge hub, facilitating translational research and the development of good practice guidelines for diagnosis and care, and supporting cross-border registries. By gathering and analyzing a large pool of patient cases, ERNs should contribute to observational studies and clinical trials, leading to new insights into RD and new drug therapies with potentially far-reaching benefits for patients.

## How does the EU promote international collaboration for rare diseases: statistical analysis

**Figure 1:**
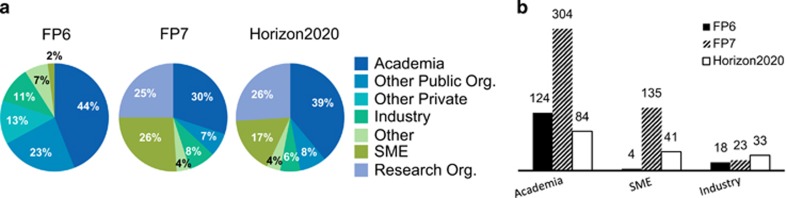
(**a**) Comparative analysis of the type of organizations supported by the EU throughout the framework programmes*. Industry: any commercial private entity larger than SME; other private: other private non-commercial organizations; other public: other public non-commercial organizations; SME: small and medium-size enterprises. (**b**) Overview of the EU support throughout the framework programmes* presented per three main organization types. The numbers shown refer to millions of euros. Legend: as the definition of organizations’ types has significantly evolved throughout the FPs, a straightforward comparison of participation between FPs is somewhat difficult. However, results for Academia, SME and Industry could be analyzed as the proportion of the financial support to these three particular types comes to 63% throughout FP6, 74% throughout FP7 and 70% throughout Horizon 2020. *Of note, analysis of FP7 projects was performed on 118 projects.

The EC, together with European Member States, have been working on ways to support international collaboration in RD research. Statistics of Framework Programme 6 (FP6), Framework Programme 7 (FP7) and Horizon 2020 provide insight into how various aspects of the international collaboration were supported by EU-level public funding. First, the diversity of types of organizations supported by the EU throughout the Framework Programmes: Figure 1a shows the percentage of participation of the types of organizations involved in FP6 (*n*=468), in FP7 (*n*=704) and—until January 2017 (*n*=322)—in Horizon 2020. Figure 1b shows the EU funding received per three main organization types.

Second, a landscape of the countries that were most frequently participating in FP7 and active in RD research emphasizes how important it is to promote international collaboration (Supplementary Figure 1). Throughout the FPs, countries of the EU-15 (that is, EU Member States before 1 May 2004) have always been the biggest recipients and users of the EU support, with allocated EU funding of 90% in FP6, 88% in FP7 and 95% in Horizon 2020. The distribution pattern amongst other countries is as follows: Associated Countries were allocated 6, 8 and 1% of the total EU funding in FP6, FP7 and Horizon 2020, followed by countries of the EU-13 (that is, EU Member States joining after 1 May 2004) with 3%, 2 and 2% in FP6, FP7 and Horizon 2020, respectively, and Third Countries (that is, non-EU, non-associated countries) with 1, 2 and 2% in FP6, FP7 and Horizon 2020, respectively. A variety of reasons outside the scope of this paper may account for these results. For example, this may indicate differences in infrastructure and financial resources available for RD research, although a lack of resources does not always correlate to a lack of activities.

In Supplementary Table 2, the overview of EU contribution per beneficiary country reveals an active club of five EU Members States throughout the three FPs presented, consisting of Germany, France, United Kingdom, Italy and The Netherlands. As a rule, a minimum participation of three legal entities from three Member States or associated countries is requested for a collaborative project to be eligible for potential funding. Of note, Switzerland would not appear as beneficiary of the Horizon 2020 program until 31 December 2016, except for potential grants from the European Research Council. The participation of Third Countries in RD research during FP7 included the United States, Australia, Canada, New Zealand, United Arab Emirates, Uzbekistan, Kazakhstan, China, Japan and India. Up to now, in Horizon 2020, the third countries participation includes the United States, Canada, Republic of Korea, South Africa, Senegal and Mozambique.

Finally, funded projects foster a variety of medical domains and therapeutic areas. Within FP6, the 59 projects addressing RD covered essentially the following research themes: cardiovascular diseases, diabetes, diagnostics, diseases of the nervous system and cancer. Within FP7, 118 projects addressed neurodegenerative and neuromuscular disorders, pediatric and adult cancers, immunological disorders and immune deficiencies, respiratory disorders and metabolic disorders. In Horizon 2020, the therapeutic areas cover certain infectious diseases in addition to the ones covered in FP7.

The impact of EU funding and how international collaboration has been fruitful is highlighted by the preliminary outcomes of FP7 based on the first 66 projects completed: there are clear benefits in terms of number of spin-off companies (*n*=7), number of new patent applications (*n*=43) and number of publications (*n*=1989). Of interest, a vast majority of finished projects (76%) prepared peer-reviewed publications, with many publications in high quality journals such as Nature, Nature Genetics, Nature Immunology, Nature Medicine, Cell, New England Journal of Medicine, EMBO Molecular Medicine and Journal of the American Medical Association.

## E-Rare: the European research area network for research programs on rare diseases

As only a few European countries fund research on RD through specifically dedicated national programs, the funding of collaborative research through JTCs was seen as the most effective method to enhance co-operation among scientists, thus reducing fragmentation of RD research in Europe and beyond. This resulted in the implementation of the ERA-Net E-Rare in 2006, co-financed by the EU. Its goal was to foster collaborative funding of relatively small and focused research consortia, and enhance complementarity to the larger multinational groups usually funded by the EU.

At the start of E-Rare-1 in 2006, the partnership consisted of eight countries but the focus and reach of E-Rare have evolved. It began with activities aimed at enhancing the knowledgebase of the European RD research landscape as well as issuing two transnational calls in 2007 and 2009. The second phase of E-Rare (2010–2014) included 15 countries and annual calls for proposals. During this time, the focus was on deepening the co-operation and co-ordination among the E-Rare partners by enabling the systematic exchange of information on national programs and by implementing strategic activities aimed at sustainable development and extension of the network of RD research funders.

Currently, E-Rare-3 is composed of 26 public bodies, ministries and research funding organizations from 18 countries: Member States (Austria, Belgium, France, Germany, Greece, Hungary, Italy, Latvia, Poland, Portugal, Romania, Spain and the Netherlands), three Associated Countries (Switzerland, Israel and Turkey) and two Third Countries (Canada and Japan).^8^ Through its easy-to-access transnational calls, E-Rare has provided a successful platform for entities interested in collaborative transnational research funding. The participation of national funding agencies in E-Rare Joint Transnational Calls (JTCs) is an effective tool to increase interoperability of funding practices and programs. Continuous co-operation of funding organizations, particularly for the implementation of JTCs, influences the alignment of processes, funding procedures and timelines of JTCs in addition to the gained common knowledge. The high number of proposals received in response to the JTCs of E-Rare highlights the great potential and diversity of the European RD research community and the need for funding of collaborative projects. Special consideration is given to avoid duplication with Horizon 2020 calls in the area of RD.

In addition to the annual transnational calls, the collaborative approaches now extend to the relevant European Research Infrastructures with the aim to customize their services to the demand of RD researchers. The long-established collaboration with EURORDIS-Rare Diseases Europe has been further strengthened by the development of new funding models and the involvement of patient organizations in research. In 2012, E-Rare also became a member of the International Rare Diseases Research Consortium (IRDiRC) and strongly contributes to its ambitious objectives (see below).

## How does E-Rare promote international collaboration: statistical analysis

Since 2006, E-Rare has launched eight JTCs for projects, investing over €92 million in research on RD. Exceptionally, in 2015, the EC contributed to funding of research projects. The competitive nature of the JTCs has resulted in the funding of high quality projects. A large proportion of submitting researchers have outstanding track records with publications in high impact journals. 498 scientific teams compose 106 funded consortia. The multinational projects involve a range of three (mandatory) to ten research groups. Interestingly, the collaborations follow geographical, historical and linguistic proximity (Supplementary Table 3). In order to increase the level of collaboration and enhance the transfer of knowledge in specific regions, in 2015, E-Rare decided to encourage the inclusion of research teams from Eastern European countries (EEC; Hungary, Latvia, Poland, Romania and Turkey). This resulted in eight projects with EEC components, significantly increasing their success rate.

**Figure 2:**
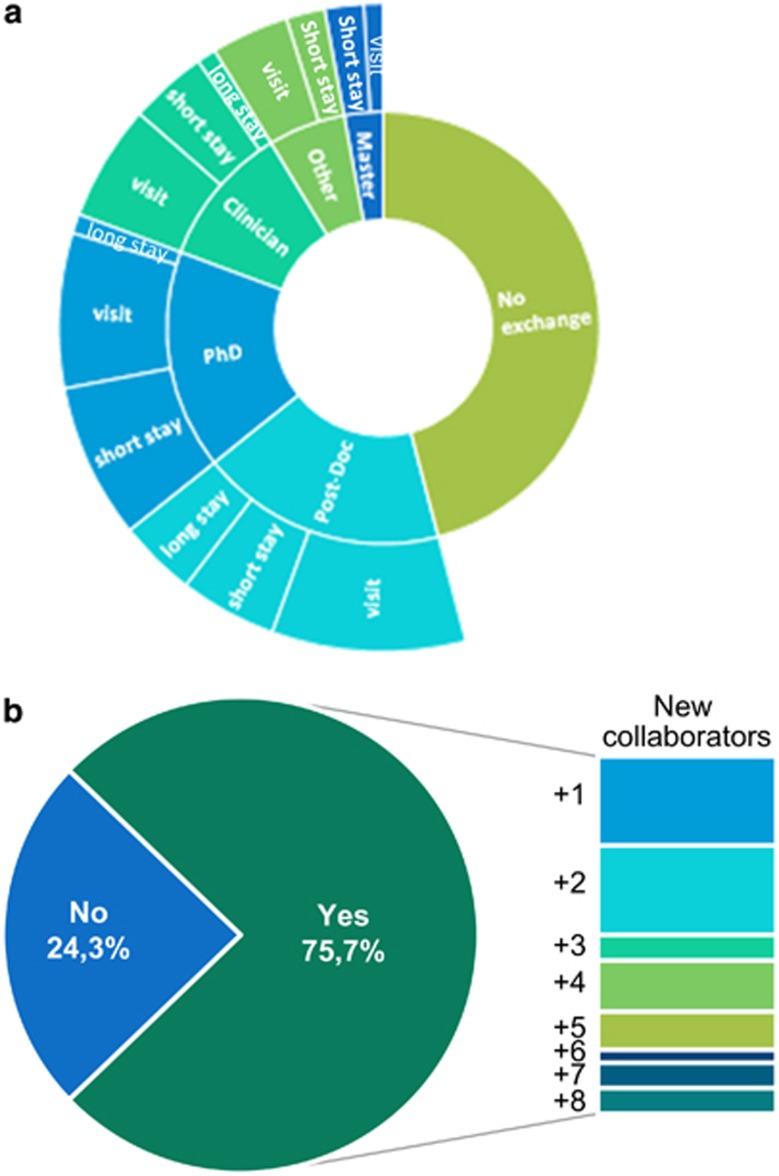
(**a**) Exchange of researchers in E-Rare funded projects. Analysis of 37 projects financed in 2007, 2009 and 2011. Visit refers to max 2 weeks; Short stay refers to 1–12 months; Long stay refers to more than 1-year stay. (**b**) Establishment of new collaboration during the lifetime of the research project. 37 projects were analyzed of which 28 developed new collaborations. Half of these new partnerships involves 1 or 2 new partners. The other 14 consortia developed collaborations with more than 3 new partners during the lifetime of the project.

The transnational collaboration within projects is reflected at different levels. One of the most important aspects is the training and exchange of personnel. The analysis of 37 projects completed since 2007 showed that, in total, 83 MSc students and 108 PhD were trained. In addition, at least half of the projects reported an exchange of students, PhD and post-docs, in either short- or long-stays (Figure 2a). A survey, launched to monitor the sustainability and broadening of collaborations, showed that 77% of consortia continue their co-operation after the close of the projects. This was also true for transnational partnerships established during the application phase for an E-Rare call even if they did not succeed in obtaining funding. Within 37 financed consortia analyses, 28 established new collaborations during the course of the project (Figure 2b), which brought additional expertize and enriched the outcomes. E-Rare is thus considered as ‘stimulator’ of transnational, enduring partnerships. Moreover, researchers reported that participation in E-Rare projects served as preparation for larger transnational partnerships such as those required for calls of the EC. Thereby, 55% of E-Rare consortia succeeded in obtaining subsequent European funding. This confirms the achievement of E-Rare’s initial goal of funding smaller partnerships complementary to EU-funded consortia. Finally, the number of joint publications also confirmed this success: 525 peer-review articles were published with a mean impact factor of 7.32 (range from 0.5 to 59).

The topics of E-Rare calls are broad and cover the whole range of medical domains. In all calls, the projects have to involve a group of RDs or a single RD following the European definition, that is, a disease affecting not more than five in 10 000 persons in the European Community, EU-associated states and Canada. In all calls (except for the JTC 2016), interventional clinical trials were excluded due to budget limitations of national funding agencies. Furthermore, research concerning rare infectious diseases, rare cancers and rare adverse drug events in treatments of common diseases were also excluded in the past joint calls. When necessary, and based on the consultation of RD research community, the so-called ‘focused’ calls are implemented. As a result, in 2012, E-Rare launched a call dedicated specifically to young investigators. In 2014, the call focused on new therapeutic approaches and, in 2016, the goal was to finance clinical trials using repurposed molecules to treat RD.

**Figure 3:**
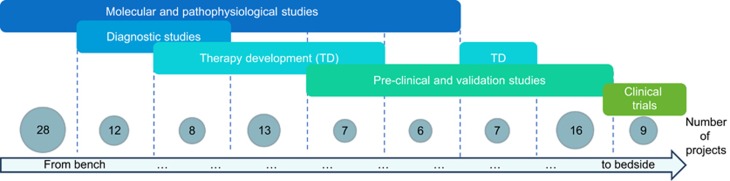
Research pipeline of projects financed by E-Rare. Analysis of all projects funded between 2007 and 2016. The projects funded by E-Rare are covering the whole research pipeline from molecular and pathophysiological studies to clinical trials. All research projects have been analyzed to classify their work in 5 categories (non-exclusive): molecular and pathophysiological studies; diagnostic studies; new therapeutic approaches; pre-clinical and validation studies; clinical trials. However, many of them include studies that could be assigned to more than one category. The numbers represent therefore projects reflecting one or combination of categories. As example: 28 projects focus on molecular and pathophysiological mechanisms of RD (only), whereas 8 projects include studies from molecular and pathophysiological mechanism to the set-up of new therapeutic approaches and diagnostics.

All medical domains are represented in projects submitted and financed (Supplementary Figure 2). The biggest share (24.2% of submitted projects) corresponds to neurology, which reflects proportionally the size of the community focusing on this domain. In terms of success rate, the most rewarded are neurology and immunology projects, but recently applications in cardiology have been increasing. Most importantly, E-Rare calls respond to the coverage of the whole bench-to-bedside pipeline. As shown in Figure 3, the 106 projects expand from molecular and pathophysiological studies, to diagnostics and therapy development and clinical trials. Noticeably, many of the funded projects cover more than one approach. This reflects the interdisciplinary nature of research consortia that combine expertize and access to technology available in different European and international laboratories.

One of the most important goals of E-Rare funding schemes is to allow transnational collaborations that foster pooling of scarce resources, access to patients and knowledge. The ex-post analysis of 37 projects financed between 2007 and 2011 showed that all of them were able to achieve critical mass of resources necessary to complete their goals. In addition, 26 of the consortia demonstrated intensive exchange of samples as well as creation of databases (17 projects), biobanks (14 projects) or registries (8 projects; Supplementary Figure 3). These ‘infrastructures’ were created either from scratch (42.1%) or by pooling existing, but disconnected sources (57.9%). More detailed surveys revealed that generation of such resources within research projects is not always optimal as their dissemination and sustainability may not be possible.

## Other means of support: co-operation with European research infrastructures and patient organizations

Although the RD research community has already recognized E-Rare as a collaboration ‘stimulator’ through its funding activities, the Consortium decided to take further steps towards enhanced and efficient collaboration between scientists and other relevant stakeholders. Consolidated interactions with the European Research Infrastructures (RIs) and Patient Organizations (POs) are part of this strategy.

### Collaboration with European Research Infrastructures

The European Strategy Forum on Research Infrastructures was established and mandated by the EU Council in 2002 with the aim of supporting a strategic approach to the development of pan-European RIs to meet the long-term needs of the research communities across all scientific areas.^9^ These infrastructures refer to major equipment, resources (for example, collections, archives and scientific data) or e-infrastructures (for example, computing systems or communication networks). This consolidation of knowledge and resources lying in different countries would enable the progression of high-caliber science and technology and facilitate knowledge sharing and innovation.

At present, 14 European RIs dedicated to health & food domains are operational.^10^ Out of them, 11 are particularly relevant for biological and medical sciences and seven were chosen by E-Rare as most pertinent for RD research: (i) Biobanking and BioMolecular Resources Research Infrastructure (BBMRI), (ii) European Advanced Translational Research Infrastructure in Medicine, (iii) European Clinical Research Infrastructure Network (ECRIN), (iv) Distributed Infrastructure for Life-science Information (ELIXIR), (v) European Infrastructure of Open Screening Platforms for Chemical Biology (EU-OPENSCREEN), (vi) Integrated Structural Biology Infrastructure (INSTRUCT) and (vii) European Research Infrastructure for the Generation, Phenotyping, Archiving and Distribution of Mouse Disease Models (INFRAFRONTIER).

The analysis of collaborative research projects submitted and financed by E-Rare revealed that 98% of them use services/infrastructures like biobanks, data repositories, imaging, high throughput sequencing or screening and animal model facilities. This was also confirmed by the outcomes of funded projects where creation or use of existing data and registries together with extensive exchange of samples and animal models were reported regularly. However, only a few projects indicated use of the European RIs, putting in question the origin of applied standards as well as sustainability of generated results. Based on these inputs and corresponding feedback from the E-Rare funding bodies, it was agreed that close collaboration with the European RIs should both mitigate the problem of discrepancies in practiced standards, and ensure the dissemination and sustainability of outcomes especially biobanks, registries, databases or models.

In order to facilitate the access of RD research community to those initiatives, a comprehensive list and dedicated thematic pages were created on the E-Rare website (http://erare.eu/infrastructures). Moreover, since 2015, E-Rare encourages the use of European RIs within its calls for transnational projects. In 2016, an explicit pilot action was implemented in collaboration with ECRIN where applicants using ECRIN services could benefit from so-called ‘ECRIN on board’: free-of-charge assistance in the preparation of their proposal to the E-Rare call. Among 30 projects submitted, 11 included collaboration with ECRIN, eight passed the pre-selection stage and four of them were eventually granted (out of eight recommended for funding) ensuring 36.4% success rate (in comparison with 22% success rate of projects not using ECRIN). In this specific case, the added value of using the European Research Infrastructure reflected, as expected, the quality and knowledge of the management of transnational clinical trials that is particularly complex. Thus, the alliance of dedicated institutions from different countries gathered into one infrastructure can directly target the issues and bring added value to the transnational consortium of researchers.

Importantly, the efforts to integrate European RIs into the practice of RD research are not unidirectional. The infrastructures themselves implemented a series of projects and tools aimed at particular needs of the RD research community. On that account, projects like CORBEL,^11^ ADOPT-BBMRI ERIC^12^ or ELIXIR-EXCELERATE^13^ led by major RIs integrate tasks dedicated to facilitated and targeted user access, collection of samples, promotion of harmonized standards, and creation of a registry of data resources and analysis tools critical for the development of RD research.

### Collaboration with patient organizations

Another important dimension of support to transnational collaboration in RD research is the engagement of patients. A study of EURORDIS-Rare Diseases Europe showed that 70% of POs not only finance but also initiate research projects. In addition, patients are interested in and concerned by all areas of research including basic, clinical, genetics, therapeutics and social science.^14^

**Figure 4:**
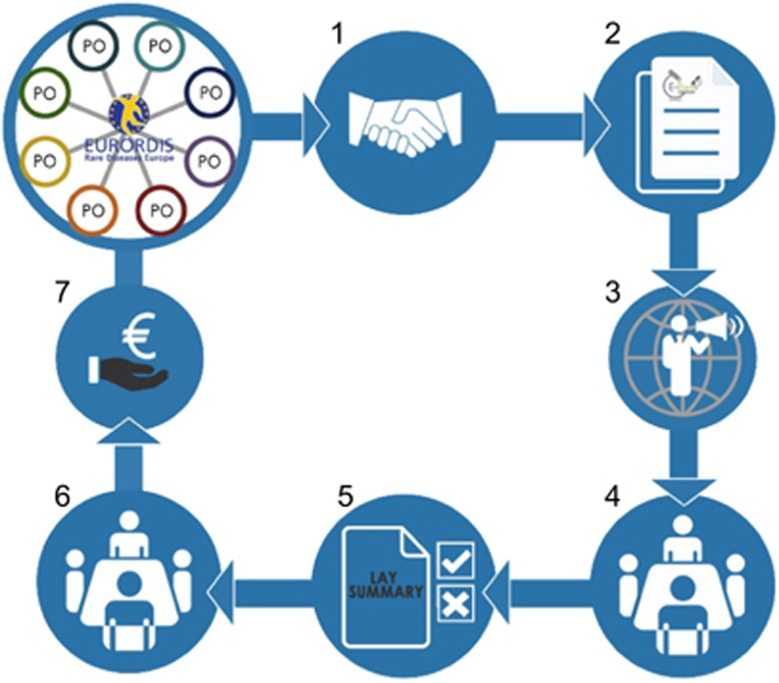
Schematic presentation of involvement of Patients’ Organizations in E-Rare funding activities. The process is divided into following phases: 1, expression of interest; 2, preparation of call documents; 3, call launch and advertisement; 4, 1st scientific evaluation; 5, PO’s choice of projects of interest; 6, 2nd scientific evaluation; 7, common funding decision.

Although patient representatives participated in E-Rare since its inception, their role was confined to advisory functions. In March 2014, EURORDIS-Rare Diseases Europe launched a survey in which 60 POs (out of which 44 had funding capabilities) expressed their interest in participating actively in E-Rare and a strong willingness to collaborate with researchers, including providing logistical and financial support. Since some of the E-Rare funding organizations already had experience co-funding research with POs, it was agreed that, with the support of EURORDIS-Rare Diseases Europe, a new model of collaboration between funders and POs would be established. The goal was to engage patients, in a transparent manner, at all levels of research funding: from definition of topics, through evaluation of projects, to budgetary investment (Figure 4). In addition, applicants were encouraged to involve patients in their proposals. As a result, almost all projects submitted to the E-Rare call in 2016 involved patients as contributors or partners. From the funders’ side, six POs decided to co-finance some of the selected projects. The participation of patients not only allowed better design of research proposals (as revealed in the evaluation process), but also enabled better recruitment of relevant cases and the extension of collaboration to countries not participating in the call. This positive experience will be further pursued in order to strengthen the researcher—patient—funder triangle.

## National vs international collaboration in rare diseases research in Europe

Orphanet is the most comprehensive source of information, in terms of geographical and domain coverage, specific for RD worldwide.^15^ It is co-financed by Member States and the EU. It comprises 40 countries in which Orphanet experts collect, annotate and classify (according to the Orphanet nomenclature) information on RD. This includes data on expert centers, patient organizations, diagnostic laboratories, research projects, clinical trials, registries, databases and infrastructures for research.

The classification of research projects is performed according to their main objectives, and those qualifiers can be grouped in the following categories: basic research, pre-clinical research and observational/epidemiological/natural history studies. In addition, information on the origin of the projects (national or multinational), related source of funding (funding bodies), as well as starting and end dates are collected.

Data on 3052 research projects started or ongoing from 2010 on was collected. Importantly, 14% of them are international collaborations funded mainly funded by the EU and/or through the E-Rare mechanism. This confirms that, at present, EU and E-Rare schemes ensure major support of RD transnational research projects in Europe although 86% of RDs research funding remains national. Interestingly, the comparison of national and international projects revealed that the latter focus on pre-clinical research (40 vs 20% for national) while national projects cover mostly basic research approaches (67%).

Gene therapy development represents a substantial part (22–24%) of pre-clinical research studies, and this proportion does not differ between national and transnational studies. The three main medical domains in which these projects focus on are neurology, immunology and metabolic diseases. Transnational pre-clinical gene therapy projects are devoted to rare immune diseases (24%) followed by neurological diseases (20%) and inborn errors of metabolism (12%), whereas rare neurological diseases predominate in national gene therapy projects (36%) followed by metabolic diseases (16%) and immune diseases (7%).

## Examples of successful rare disease-related collaborative gene therapy projects

### Projects financed by the EU

The EU had supported during FP6 a €12 million European network of excellence for the advancement of clinical gene transfer and therapy (CliniGene^16, 17^) involving 42 participants from 14 countries. Its role was to foster interaction between all stakeholders—regulators, pre-clinical and clinical investigators, scientists, companies and patient representatives—in order to streamline integration of multidisciplinary expertize and generate new knowledge; to establish the quality, safety, efficacy and ethically acceptable standards for clinical gene transfer products; and to identify and accelerate the ‘critical path’ from pre-clinical to clinical phase. One particular feature of this network, organized into seven technology platforms, was the possibility to fund its own internal collaborative and exchange program that could target specific bottlenecks such as establishment of improved producer cells, development of validated production and purification protocols for viral and non-viral vectors, address the ethical, legal and regulatory component and facilitation of clinical trials for rare or common diseases (cancers, adenosine deaminase severe combined immunodeficiency, Leber congenital amaurosis, Parkinson’s disease or X-linked adrenoleukodystrophy). The main results of CliniGene were: integration of EU-wide research and facilities in the field of gene therapy; overcoming of most scientific bottlenecks identified and targeted by the seven technology platforms; publication of more than 745 papers; financial support to over 90 projects targeting novelty and international collaborations between 45 laboratories and companies; creation of new improved producer cells; development of highly efficient production and purification for most important viral and non-viral vectors; implementation of a reliable and validated vector biosafety platform addressing safety and efficiency *in vitro* and *in vivo*; contribution to the ethical, legal and regulatory components of these new therapies to the European Medicines Agency and facilitation of clinical trials performance; training as well as technology transfer to industry.

During the FP7, several clinical trials for RD were supported by the European funds. As example, the Skip-NMD project^18^ (10 partners from five countries) aims to restore dystrophin production in a subset of Duchenne muscular dystrophy boys by administration of a drug, which ‘skips’ the mutations causing Duchenne muscular dystrophy. The presence of the US-based Sarepta Therapeutics in the consortium led to the expansion of the initial clinical trial and provided expertize in bringing an oligonucleotide to the market.^19^ The project is a follow-up to previous EU projects on neuromuscular dystrophies. In addition, the partners also co-ordinate the worldwide endeavors of several laboratories developing standardized methods of dystrophin quantification, which could be used as an outcome measure in clinical trials involving Duchenne muscular dystrophy patients.

Amongst the ongoing Horizon 2020 projects, the SCIDNET consortium^20^ (12 partners from six EU countries) deals with multi-center lentiviral-based gene therapy trials for severe combined immunodeficiency diseases: adenosine deaminase severe combined immunodeficiency, Artemis and SCID-X1, and also derives from previous successful collaborative EU grants. Importantly, for adenosine deaminase severe combined immunodeficiency, the planned clinical trial should lead to marketing authorization of the gene therapy product as a licensed medicine.

### Projects financed by E-Rare

(All projects cited can be found on *http://www.erare.eu/all-funded-projects*).

**Figure 5:**
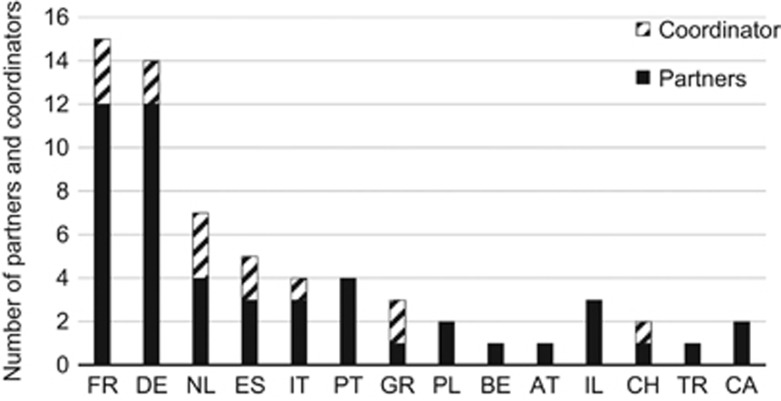
Coordinators and partners in E-Rare gene therapy research projects by country (International Organization for Standardization abbreviation is used).

In seven out of eight E-Rare calls, research teams could apply for pre-clinical studies on gene therapy for RD; the focus of JTC 2016 was on clinical research of repurposed molecules for RD and therefore no applications for gene therapy research could be submitted. Analysis of the 98 projects funded in the first seven calls showed that 14 projects were at least partially dedicated to gene therapy approaches. In all E-Rare calls, with the exception of JTC 2007 and JTC 2013, projects focusing on gene therapy were selected for funding. Sixty-four research groups composed these 14 financed research consortia (Figure 5). Interestingly, some countries concentrate more research groups on gene therapy approaches than others. The participating teams were located in research institutes, universities, hospitals or blood banks. In one project (Drug_FXSPreMut) a company is involved.

Five projects were fully dedicated to gene therapy approaches: GETHERTHAL, GETHERTHALPLUS, Cav4MPS, TRANSPOSMART and SpliceEB (Supplementary Table 4). In nine projects gene therapy approaches composed a part of the scientific approach. Noticeably, the GETHERTHAL project financed in 2011 obtained a subsequent funding from E-Rare in 2015. The medical domains in which gene therapeutic approaches were investigated were blood diseases (three diseases in five projects), two metabolic diseases (two projects), two immune deficiencies (two projects), lymphatic disease, skin disease, deaf—blind disease, neuromuscular disease and neurodegenerative disease.

In their funded projects research groups aim to improve current vector technology, improve transfer of nucleic acids for therapeutic purposes, develop novel strategies for cell or organ targeting or assess the feasibility of the gene therapeutic approach in appropriate cell models and animal models (for example, in the projects GETHERTHAL, EURO-CGD, HEMO-iPS, TRANSPOSMART and SPLICE-EB). In the project CAV-4-MPS, intracranial administration of helper dependent canine adenovirus type 2 vector coding for the missing enzyme in mucopolysaccharidosis VII resulted in short and long-term expression of the therapeutic enzyme in an mucopolysaccharidosis VII mouse and an MPSVII dog model, resulting in global biochemical correction in the brain. In the project Theralymph, several therapeutic approaches for treatment of hereditary lymphedema are investigated: pharmacological and tissue engineering next to gene therapeutic approaches.

## Outreach beyond Europe: international rare diseases research consortium

The IRDiRC was created in 2011 to facilitate co-operation and collaboration on a global scale among the many stakeholders active in RD research, to maximize the output of RD research efforts around the world.

IRDiRC was originally conceived in 2009 by the Directorate General of Research and Innovation for the EC and the Director of the US National Institutes of Health (NIH), who envisioned speeding progress in RD research by coordinating their RD research funding. Over the course of the first preparatory workshop in Reykjavík, Iceland in 2010, it became clear that the success of the initiative would be dependent on the integration of the activities of not just funding agencies, but also academic researchers, companies, regulatory agencies and patient advocacy organizations.^21^ As such, membership of IRDiRC was expanded to include those constituents.

IRDiRC aims to facilitate the achievement of two overarching objectives by the year 2020: to contribute to the development of 200 new therapies and the means to diagnose most RDs. The crucial outcome of this work will be improved health through better diagnostic capabilities and novel therapies for people living with RDs throughout the world.

**Figure 6:**
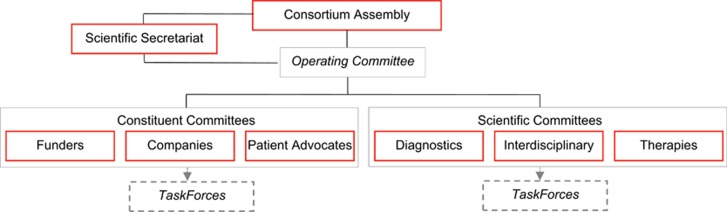
IRDiRC governance structure.

Organizationally, IRDiRC is comprised of a Consortium Assembly, an Operating Committee, three Constituent Committees (Funders, Companies and Patient Advocates), three Scientific Committees (Diagnostics, Interdisciplinary and Therapies), Task Forces and the Scientific Secretariat (Figure 6). Representatives of all member organizations of IRDiRC form the Consortium Assembly, which focuses on information exchange and development and co-ordination of scientific and policy efforts that advance IRDiRC goals. The Operating Committee manages the preparation and advancement of IRDiRC activities, processes information and enables more effective management of the Consortium. The Constituent Committees co-ordinate activities, identify roadblocks to progress and designate priorities in their respective constituency area. The Scientific Committees advise the Consortium Assembly on research priorities and progress, identify gaps where research funding is required, encourage exchange of best practices and agree on actions to reach IRDiRC goals in their scientific area. The Task Forces are time-limited and tackle specific topics identified by the Committees as important to advancing IRDiRC goals. The Scientific Secretariat—supported by a contract awarded by the EC to the French National Institute of Health and Medical Research (INSERM, US14)—supports co-ordination between and amongst all members, tracks activities and progress made in the field of RD research, and contributes to the timely achievement of IRDiRC goals.

### IRDiRC policies & guidelines: establishing a common platform for progress

IRDiRC focuses on strengthening international co-operation to bring about rapid progress in the field of RD research. Co-ordination of efforts that address common roadblocks is key to maximizing the collective impact of global investments in RD research and accelerating progress. To guide its work, IRDiRC developed a set of Policies and Guidelines, which are the principles that IRDiRC members agree to follow, focused on the following areas: data sharing and standards, ontologies, diagnostics, biomarkers, patient registries, biobanks, natural history, therapeutics, models, publication and intellectual property, and communications about the Consortium.^22^

### IRDiRC as a solution driver: Task Forces, ‘IRDiRC recognized resources’

The members of IRDiRC support research projects that contribute to the Consortium objectives and goals. A number of Task Forces have been created by the Scientific and Constituent Committees to identify limitations to efficiency and effectiveness in global RD research, suggest and/or create solutions including standards and tools, and publish recommendations. One of the earliest Task Forces initiated was a collaboration with the Global Alliance for Genomics and Health (GA4GH) and others to create Matchmaker Exchange, which enables RD gene discovery through a federated network of genotype and rare phenotype databases.^23^ The International Consortium of Human Phenotype Task Force developed standards for interoperability among databases which has enabled the linkage of a variety of RD phenotype and genotype databases. The Patient—Centered Outcome Measures Task Force and Small Population Clinical Trials Task Force investigated aspects of RD clinical studies and produced recommendations to, respectively, (1) enable the development of clear outcome measures that benefit patients,^24^ and (2) advance discussions on optimizing efficient and innovative trial designs relevant to small populations.^25^ Similarly, the Data Mining and Repurposing Task Force made recommendations on strategies for optimizing the capture, sharing and integration of research and patient data to further research and development investments and realize the full potential of data mining and drug repurposing approaches (draft in review). Another joint IRDiRC-GA4GH effort was the Automatable Discovery and Access (ADA) Task Force that developed an ADA matrix which provides a standardized way to represent consent and other conditions of clinical data use, making the information computer-readable and available for automated search and sharing activities (software available to download and use—https://genomicsandhealth.org/work-products-demonstration-projects/automatable-discovery-and-access-matrix).^26^ The Privacy-Preserving Record Linkage Task Force, also a joint effort with GA4GH, is developing guidelines on the ethical, legal and technical requirements of participant identifiers in RD research. In addition, they are investigating a technical solution for a system to de-duplicate and link data sets for use by researchers without knowledge of participants’ identity, and to enable the potential re-identification of participants for clinical purposes. Among the additional Task Forces due to commence shortly are those addressing approaches for investigating diagnostic cases unsolved via current technologies, addressing bottlenecks in data sharing including making policy recommendations around clinical data sharing, and developing best practices for patient engagement in RD research.

Like RD patients, researchers who study RDs are often few in number and geographically dispersed, impeding the ability of RD researchers—particularly those new to the field—to identify resources and tools that might facilitate their work. In order to address this need, the Consortium developed an indicator called ‘IRDiRC Recognized Resources’ to highlight publically available resources that researchers in the RD community have found useful and which, if more broadly used, might accelerate advances in RD research.^27^ To receive this designation, resources must undergo a peer-review process by IRDiRC Scientific Committee members and external experts in the field. To date, the label has been given to 13 resources: three guidelines, four platforms, two reference databases, two standards, and an advisory committee (Table 1).

### IRDiRC impact/future prospects

As IRDiRC approaches the end of its sixth year, its two main initial objectives—to contribute to the development of 200 new therapies and the means to diagnose most RDs—have largely been achieved. This presents IRDiRC with the exciting opportunity to set new and even more ambitious goals for the next phase of the Consortium. The scale of the challenges in RD research dictate the need for exponential improvement in efficiency and effectiveness of the understanding, diagnosis and treatment of RDs. IRDiRC’s new goals, currently being formulated, aim to propel such changes via co-ordination, collaboration and collective action.

## Conclusions

Collaboration is an integral part of research especially in RD where the expertize and patients are scattered. For almost two decades specific efforts have been made at national, European and international levels to strengthen co-operation not only in research, but also at regulatory, funding and health care levels. The creation of IRDiRC brought additional centralization of efforts and delineated strong goals for all relevant stakeholders. As demonstrated above, these endeavors resulted in powerful outcomes. The consistent funding provided by the EU, E-Rare and individual countries, demonstrates the power and possibility of continuous support for RD projects across the translational spectrum. Tools have been developed to facilitate the translation of results into tangible outcomes and make them more sustainable and sharable. Partnerships with European Research Infrastructures developed by E-Rare and indicated by ‘IRDiRC Recognized Resources’ are part of this effort. With the help of IRDiRC’s Scientific Committees, recommendations have been developed to (1) highlight to funders the most pressing areas that necessitate support and (2) emphasize the importance of issues like data sharing and harmonization to researchers. The involvement of patients as main actors and crucial stakeholders completes this picture.

All these efforts led to the creation of a favorable ecosystem promoting the development of both new and more sustainable collaborations, connecting researchers from internationally dispersed laboratories. The outcomes of these collaborations are tangible: faster diagnosis of RD patients through enhanced discovery of causative genes and production of relevant clinical practice guidelines; better understanding of natural history of diseases through creation of common registries and databases; accelerated creation of animal and cellular models laying the basis for research on disease mechanisms and treatment options and boosting of innovative therapeutic approaches and clinical trials for new and more suitable treatment options.

Notwithstanding, these encouraging results also illuminate new challenges that have yet to be overcome as too many RD patients continue to remain without adequate diagnosis and treatment. It is clear that many challenges still remain: (i) better understanding of RD mechanisms and faster translation of this knowledge into new diagnostics and treatments; (ii) development of novel approaches to clinical trial methodologies in small populations to facilitate the demonstration of the safety and efficacy of orphan drug candidates; (iii) improvement of health technology assessment methodologies that can be applied to the RD field and support best practices; (iv) investment in RD research databases and patient registries that are less fragmented and encourage data sharing and re-use via linking to other international platforms. In the EU Member States, ensuring and improving the equitable provision of health care on RD is also very challenging.^28^ Thus, more efforts focused on shortening the translational pipeline and closing the gap between research and care are needed. In February 2017, at the 3rd IRDiRC Conference in Paris, France, stakeholders of RD research from around the globe gathered to discuss global progress to date, transformative new approaches to RD research, and challenging goals to be set for the next decade. What is clear after the 2-day conference: the international RD research community is eager to share knowledge and experience, work collaboratively across borders and address common roadblocks to maximize the impact of investments in order to bring diagnoses and therapies to RD patients. It is time to build new bridges and raise the bar for RD research worldwide.

## Figures and Tables

**Table 1:** IRDiRC Recognized Resources

*Resource name*	*Type*	*Description*
*Facilitating international data sharing*		
International Charter of Principles for sharing Bio-specimens and Data	Guideline	The Charter provides recommendations for successful legally and ethically grounded sharing of bio-specimens and data
Framework for Responsible Sharing of Genomic and Health-related Data	Guideline	The Framework for Responsible Sharing of Genomic and Health-Related Data provides a principled and practical framework for the responsible sharing of genomic and health-related data
PhenomeCentral	Platform	PhenomeCentral is a repository for secure data sharing in the RD community, thereby connecting to other patient’s profiles
DECIPHER	Platform	DECIPHER is a database and web-based platform enabling the deposition, analysis and sharing of phenotype-linked plausibly pathogenic variation in patients with rare genetic disorders
		
*Knowledge organization and ontologies*		
Orphanet	Reference/database	Orphanet is a reference portal for information on RDs and orphan drugs
OMIM	Reference/database	Online Mendelian Inheritance is a database of human genes and genetic phenotypes comprised of over 23 000 structured free-text entries
Orphanet Rare Disease Ontology	Platform	Orphanet Rare Disease Ontology provides a structured vocabulary for RDs thereby aiming to define relationships between diseases, genes and other features of interest
Human Phenotype Ontology	Standard	Human Phenotype ontology provides a standardized vocabulary of phenotypic abnormalities encountered in human disease
International Consortium of Human Phenotype Terminologies	Standard	The International Consortium of Human Phenotype Terminologies provides the community with a set of terms to describe phenotypic features to be used by any terminologies to achieve interoperability between databases, in particular to allow the linking of phenotype and genotype databases for RDs
		
*Networking patient registries*		
TREAT—NMD Patient Registries	Platform	The TREAT—NMD Patient Registries is a global network of national registries that provides a unique entry point for access to rare neuromuscular disease patients worldwide
		
*Therapeutic development*		
Standard operating procedures for pre-clinical efficacy studies	Guideline	Standard operating procedures for pre-clinical efficacy studies are a compilation of experimental protocols to measure drug efficacy in models of neuromuscular disease
Care and Trial Site Registry	Platform	The Care and Trial Site Registry aims to assist pharmaceutical industry and clinical investigators in deciding on clinical trial site location and in the identification of potential partners for future research projects
TREAT—NMD Advisory Committee for Therapeutics	Advisory Committee	TREAT—NMD Advisory is a group expert from various origins (academic, industry drug development, patient representatives and governmental representatives) that provide guidance on the translation of therapeutics programs in rare neuromuscular diseases

Abbreviations: OMIM, Online Mendelian Inheritance in Man; RD, rare disease.
