# Supplementary figures and images for: The importance of international collaboration for rare diseases research: a European perspective
Source: Gene Ther. 2017 Jul 27;24(9):562–71. doi: 10.1038/gt.2017.29 (PMC5628265; doi:10.1038/gt.2017.29)

## Slide 1
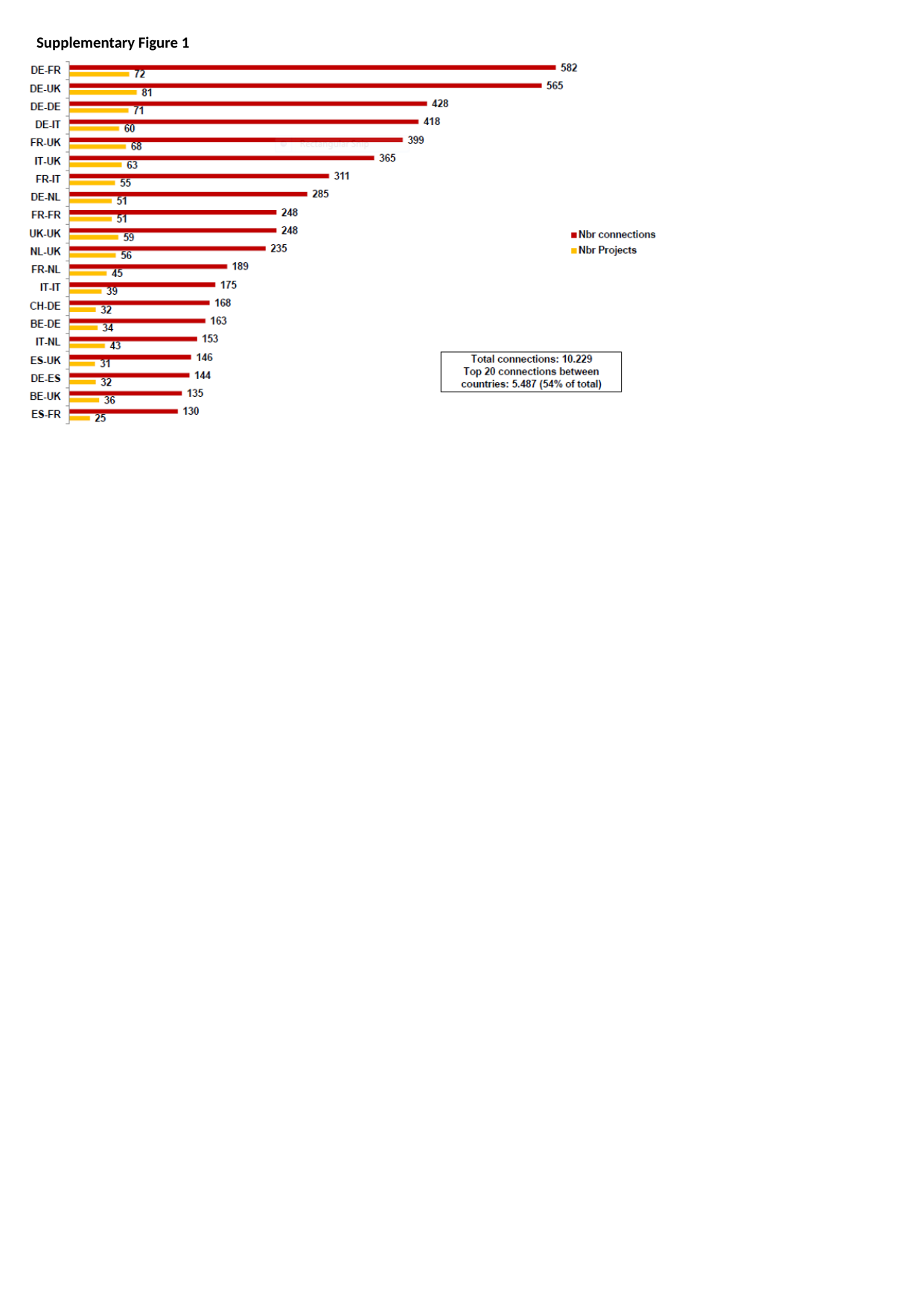

Supplementary Figure 1

Supplement: Supplementary Figure 1 [file gt201729x1.ppt]

## Slide 1
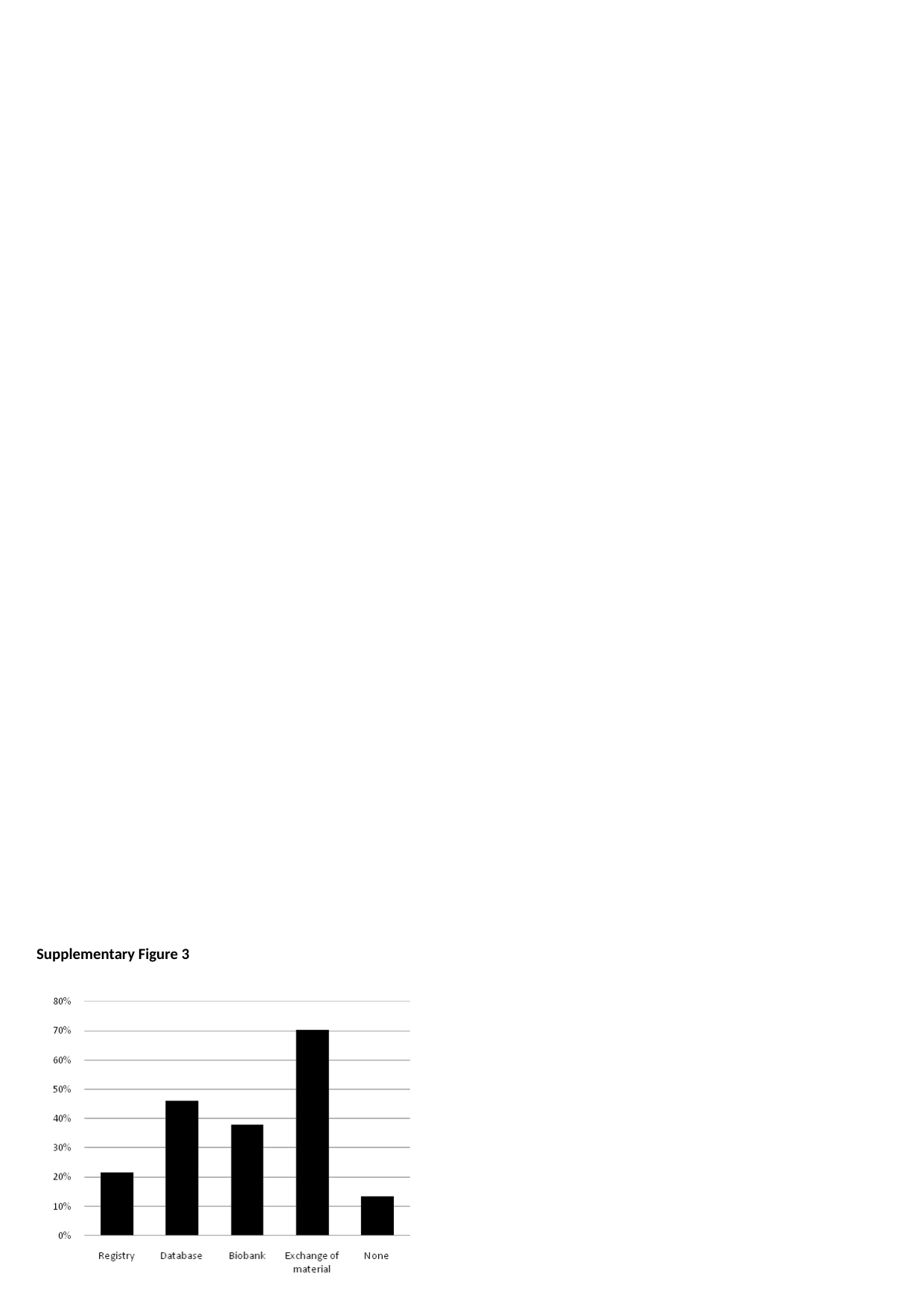

Supplementary Figure 3

Supplement: Supplementary Figure 3 [file gt201729x3.ppt]
