# Supplementary material for: The importance of international collaboration for rare diseases research: a European perspective
Source: Gene Ther. 2017 Jul 27;24(9):562–71. doi: 10.1038/gt.2017.29 (PMC5628265; doi:10.1038/gt.2017.29)
Supplement: Supplementary Figure 2 [file gt201729x2.ppt]

## Slide 1
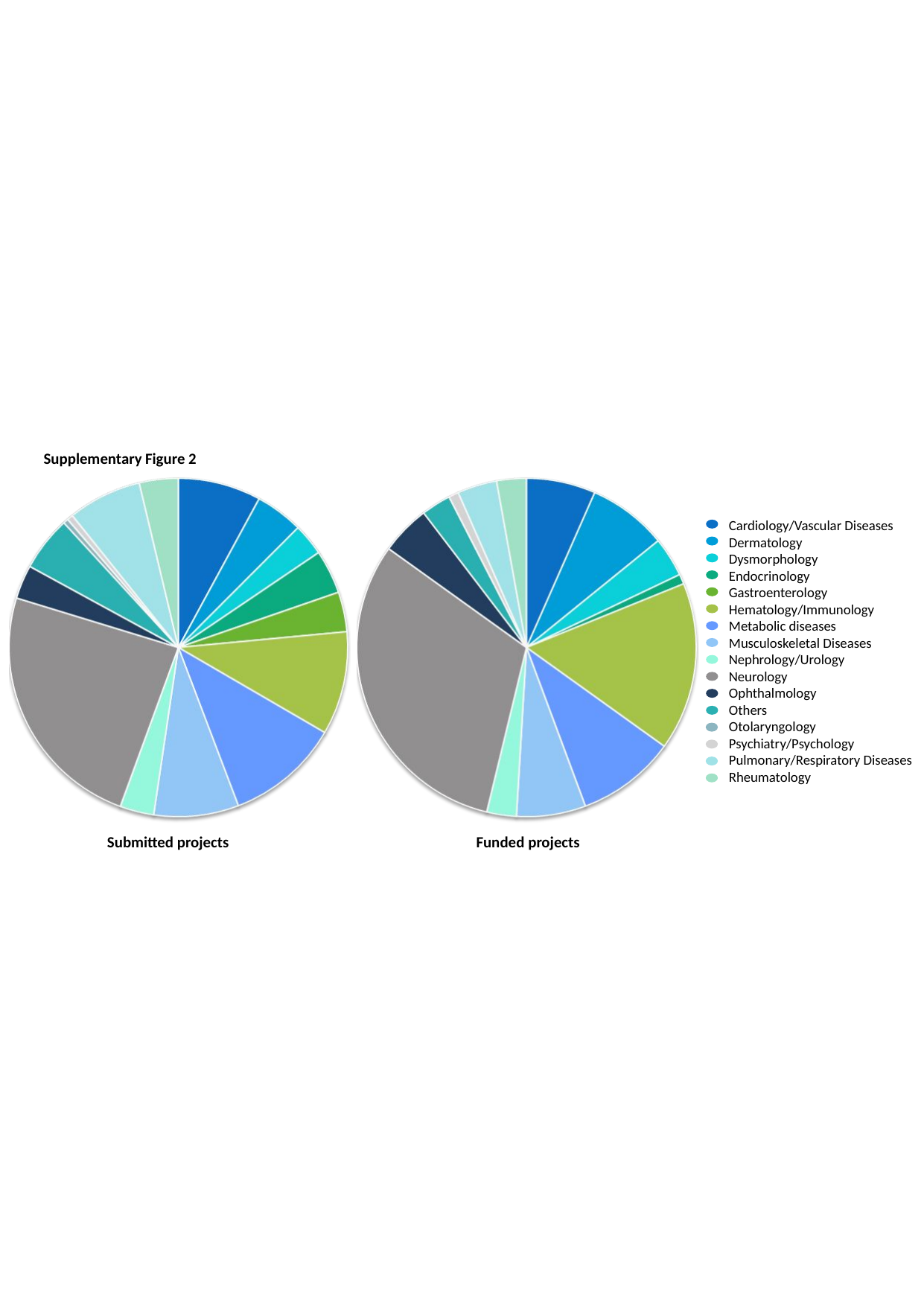

Supplementary Figure 2
Cardiology/Vascular Diseases
Dermatology
Dysmorphology
Endocrinology
Gastroenterology
Hematology/Immunology
Metabolic diseases
Musculoskeletal Diseases
Nephrology/Urology
Neurology
Ophthalmology
Others
Otolaryngology
Psychiatry/Psychology
Pulmonary/Respiratory Diseases
Rheumatology
Submitted projects
Funded projects
